# Skeletal muscle hypertrophy and attenuation of cardio-metabolic risk factors (SHARC) using functional electrical stimulation-lower extremity cycling in persons with spinal cord injury: study protocol for a randomized clinical trial

**DOI:** 10.1186/s13063-019-3560-8

**Published:** 2019-08-23

**Authors:** Ashraf S. Gorgey, Refka E. Khalil, John C. Davis, William Carter, Ranjodh Gill, Jeannie Rivers, Rehan Khan, Lance L. Goetz, Teodoro Castillo, Timothy Lavis, Adam P. Sima, Edward J. Lesnefsky, Christopher C. Cardozo, Robert A. Adler

**Affiliations:** 1 Spinal Cord Injury & Disorders Service, Hunter Holmes McGuire VA Medical Center, Richmond, VA USA; 2 Department of Physical Medicine & Rehabilitation, Virginia Commonwealth University, Richmond, VA USA; 3 Endocrinology Service, Hunter Holmes McGuire VA Medical Center, Richmond, VA USA; 4 Endocrine Division, School of Medicine Virginia Commonwealth University, Richmond, VA USA; 5 Radiology Service, Hunter Holmes McGuire VA Medical Center, Richmond, VA USA; 6 Department of Biostatistics, School of Medicine Virginia Commonwealth University, Richmond, VA USA; 7 Cardiology Service, Hunter Holmes McGuire VA Medical Center, Richmond, VA USA; 8 Division of Cardiology, Department of Internal Medicine, Pauley Heart Center Virginia Commonwealth University, Richmond, VA USA; 9 Center for the Medical Consequences of Spinal Cord Injury, James J Peters VA Medical Center, Bronx, NY USA; 10 Departments of Medicine and Rehabilitation Medicine, Icahn School of Medicine, New York, NY USA

**Keywords:** Neuromuscular electrical stimulation, Functional electrical stimulation, Resistance training, Spinal cord injury

## Abstract

**Background:**

Persons with spinal cord injury (SCI) are at heightened risks of developing unfavorable cardiometabolic consequences due to physical inactivity. Functional electrical stimulation (FES) and surface neuromuscular electrical stimulation (NMES)-resistance training (RT) have emerged as effective rehabilitation methods that can exercise muscles below the level of injury and attenuate cardio-metabolic risk factors. Our aims are to determine the impact of 12 weeks of NMES + 12 weeks of FES-lower extremity cycling (LEC) compared to 12 weeks of passive movement + 12 weeks of FES-LEC on: (1) oxygen uptake (VO_2_), insulin sensitivity, and glucose disposal in adults with SCI; (2) skeletal muscle size, intramuscular fat (IMF), and visceral adipose tissue (VAT); and (3) protein expression of energy metabolism, protein molecules involved in insulin signaling, muscle hypertrophy, and oxygen uptake and electron transport chain (ETC) activities.

**Methods/Design:**

Forty-eight persons aged 18–65 years with chronic (> 1 year) SCI/D (AIS A-C) at the C5-L2 levels, equally sub-grouped by cervical or sub-cervical injury levels and time since injury, will be randomized into either the NMES + FES group or Passive + FES (control group). The NMES + FES group will undergo 12 weeks of evoked RT using twice-weekly NMES and ankle weights followed by twice-weekly progressive FES-LEC for an additional 12 weeks. The control group will undergo 12 weeks of passive movement followed by 12 weeks of progressive FES-LEC. Measurements will be performed at baseline (B; week 0), post-intervention 1 (P1; week 13), and post-intervention 2 (P2; week 25), and will include: VO_2_ measurements, insulin sensitivity, and glucose effectiveness using intravenous glucose tolerance test; magnetic resonance imaging to measure muscle, IMF, and VAT areas; muscle biopsy to measure protein expression and intracellular signaling; and mitochondrial ETC function.

**Discussion:**

Training through NMES + RT may evoke muscle hypertrophy and positively impact oxygen uptake, insulin sensitivity, and glucose effectiveness. This may result in beneficial outcomes on metabolic activity, body composition profile, mitochondrial ETC, and intracellular signaling related to insulin action and muscle hypertrophy. In the future, NMES-RT may be added to FES-LEC to improve the workloads achieved in the rehabilitation of persons with SCI and further decrease muscle wasting and cardio-metabolic risks.

**Trial registration:**

ClinicalTrials.gov, NCT02660073. Registered on 21 Jan 2016.

## Background

Spinal cord injury (SCI) is a devastating medical condition that results in reduced aerobic fitness, glucose intolerance, and insulin resistance due to autonomic dysfunction, muscle wasting, increased regional and total body fat mass, and relative inactivity [1]. Of the more than 46,000 veterans with SCI-related disability, > 60% are overweight or obese, > 50% are glucose intolerant, and 20% are diabetic [2, 3]. There are 11,000–12,000 new cases of SCI in the United States annually. The prevalence of individuals with SCI has been estimated to be 250,000–400,000 with an estimated 14% growth in prevalence since 1988 [4, 5]. In the last two decades, costs of SCI in the United States have increased with a concomitant decline in mortality over the first year after SCI [1]. The average initial charge per case is in the range of $342,000–$1,048,000 for acute stabilization and rehabilitation, with annual charges thereafter in the range of $41,000–$182,000 [4, 6, 7]. It has been estimated that the estimated lifetime costs that result from SCI is 7.3–12 million dollars [4, 6].

The Centers for Disease Control and Prevention (CDC) and the American College of Sports Medicine have recommended 30 min of daily exercise to prevent the occurrence of health-related and obesity-related secondary complications [8]. Furthermore, guidelines were recently published by the International Spinal Cord Injury Society (ISCoS) suggesting 2–3 days per week of moderate to vigorous physical activity to lessen secondary co-morbidities [9]. However, there are several barriers that interfere with maximizing the benefits of any exercise protocol [10]. To offset some of these barriers, exercising skeletal muscle below the level of injury may enhance several cellular activities and whole-body metabolism [11–13]. Skeletal muscles serve as a large endocrine gland that controls interplay between musculoskeletal systems and other physiological systems [14–16]. Previous work demonstrated that skeletal muscle hypertrophy releases important myokines that may regulate atrophic pathways, bone, and endocrine glands. Thus, it is crucial to maintain the skeletal muscle vitality sublesionally to maintain the integrity of other physiological systems [11–13].

Functional electrical stimulation (FES) is a form of neuromuscular electrical stimulation (NMES) which provides stimulation of selected muscles in a coordinated manner resulting in a functional pattern such as walking, cycling, or rowing. This is contrary to surface NMES which is used for a single or multiple muscle groups to exercise or to move specific joint, but without the intention of producing a functional or coordinated movement [17]. FES-lower extremity cycling (LEC) is limited by the low exercise intensity, low peak oxygen uptake (0.4 L/min), and rapid muscle fatigue, which renders FES-LEC training a suboptimal modality to increase gain in lean mass and improve metabolic health after SCI [18, 19]. This may result from a mismatch when converting metabolic energy into mechanical energy.

Neuromuscular electrical stimulation (NMES) has been used to evoke exercise-induced resistance training (RT) using regular ankle weights in individuals with chronic SCI [20–24]. Several research groups demonstrated the efficacy of this approach in evoking robust muscle hypertrophy [21, 24, 25]. Dudley et al. showed that eight weeks of twice-weekly NMES RT reverted knee extensor muscle size to 75% of original size at six weeks after SCI [21]. Follow-up studies reported a 35–39% increase in muscle hypertrophy and 33% fatigue resistance of the trained knee extensors [22, 23]. Gorgey et al. noted that 12 weeks of twice-weekly NMES RT elicited an increase in skeletal muscle size of > 35%, decreased IMF and VAT by 25%, and increased insulin sensitivity and insulin growth factor-1 (IGF-1) by 25% [23]. Finally, Ryan et al. noted improvement in mitochondrial capacity by 25% following 16 weeks of twice-weekly evoked NMES RT in persons with chronic complete SCI [24]. A recent study demonstrated that 16 weeks of NMES-RT combined with testosterone replacement therapy (TRT) resulted in a 43% muscle hypertrophy of the knee extensor and 21% increase in thigh muscle cross-sectional area (CSA) accompanied with 14–17% increase in daily basal metabolic rate (BMR) compared to TRT only. [25]

**Fig. 1:**
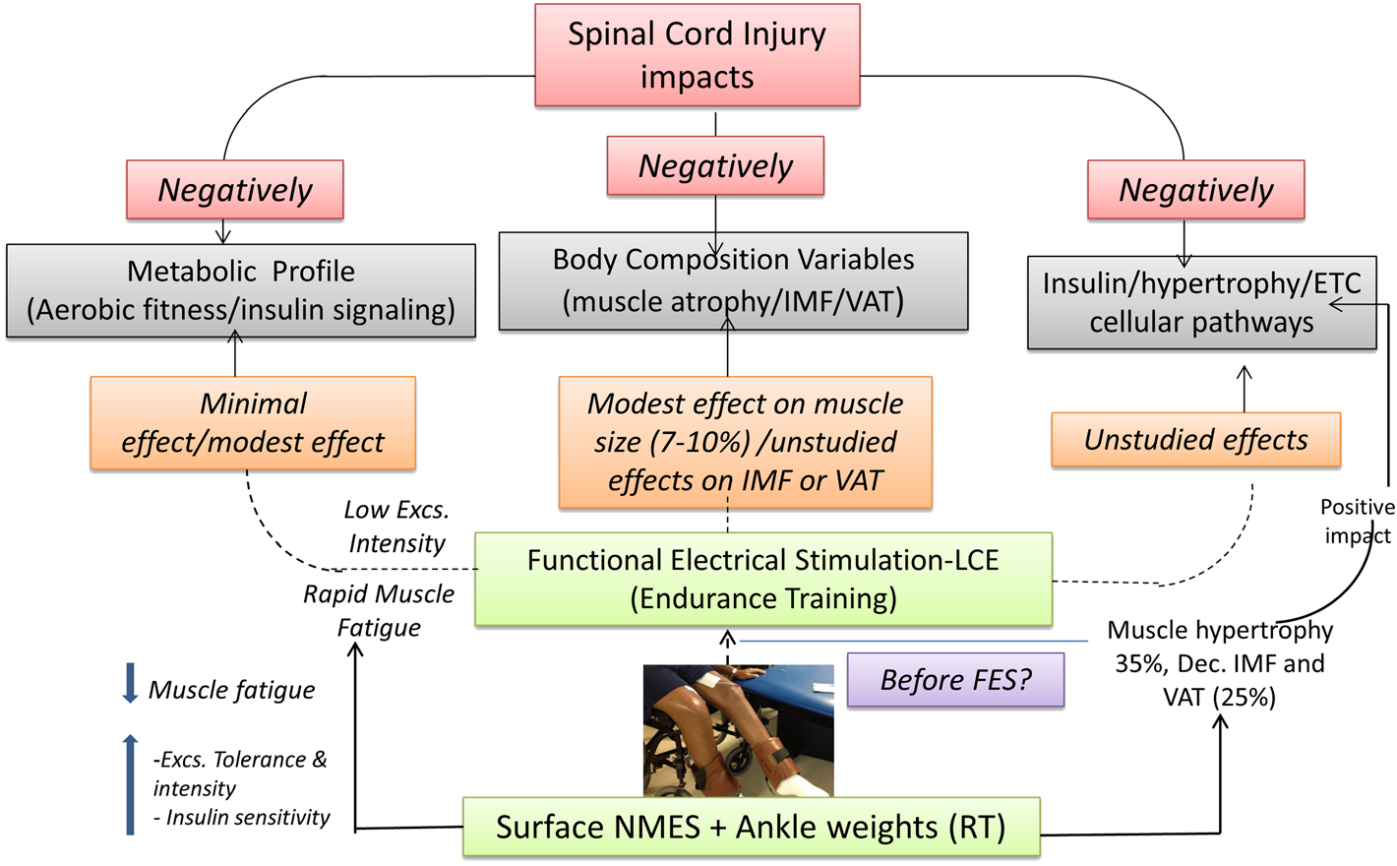
Rationale of the study. Briefly summarizes the negative impacts of SCI on body composition and cardiometabolic prolife and how adding NMES-RT to FES-LEC training may help to alleviate these health-related consequences and serve as a rehabilitation approach for persons with SCI

The objectives of the current trial are to determine the impact of evoking skeletal muscle hypertrophy using surface NMES and ankle weights before conducting FES-LEC on oxygen uptake, insulin sensitivity, and glucose effectiveness (primary endpoint variables) compared to those who undergo passive movement and FES training only. The primary question is: does evoking skeletal muscle hypertrophy lead to improvement in metabolic health benefits as outlined in Fig. 1? This prospective randomized clinical trial will also shed light on the mechanism and signaling pathways responsible for evoking hypertrophy, enhancing energy, and substrate utilization as well as improving glucose metabolism.

We hypothesize that the addition of 12 weeks of NMES combined with RT before FES-LEC will evoke skeletal muscle hypertrophy, decrease IMF and visceral fat, and enhance gains in aerobic fitness and insulin sensitivity observed during a subsequent 12-week period of exercise by FES-LEC. We also hypothesize that when NMES RT is administered before FES-LEC, the intracellular signaling related to insulin action (IRS1, Glut4) and muscle growth (Akt and mTOR) will be improved. These effects of NMES RT may further enhance the beneficial actions of FES-LEC on metabolic profile, body composition profile, and mitochondrial electron transport chain (ETC) and intracellular signaling related to insulin action and muscle growth.

## Methods

### Study design

A five-year, two-site, randomized controlled study is proposed to investigate the efficacy of NMES + FES versus Passive + FES (control group) on health-related secondary complications after SCI (Table 1). A third site will be used for protein and RNA analysis that will generated from muscle biopsy samples. Patches of muscle biopsy samples will be shipped overnight at the completion of the trial for analysis purpose (see below). All training aspects will be conducted at the SCI Exercise Body Composition Laboratory and MRI Research center located at the McGuire VA Medical Center (VAMC) and the Clinical Research Center (CRC) located at Virginia Commonwealth University (VCU). All participants will undergo metabolic studies, body composition assessments using anthropometry, dual X-ray absorptiometry and magnetic resonance imaging (MRI), and muscle biopsies. After informed consent, each individual will undergo a complete physical examination, including neurological assessment, and International Standards for Neurological Classification of Spinal Cord Injury (ISNCSCI), formerly known as the “ASIA” exam.

**Table 1:** Randomization of individuals with motor complete SCI using block randomization based on level of injury (paraplegia vs tetraplegia) and time since injury (less than or more than 10 years) into either NMES + FES or PM + FES

Subject ID	Assignment	Baseline 1	Post-intervention 1	Post-intervention 2
001–10123	PM + FES	C	C	C
002–10187	PM + FES	C	C	C
003–10122	NMES + FES	C	C	C
004–10006	NMES + FES	C	C	C
005–10128	NMES + FES	C	C	C
006–10142	PM + FES	C	C	X
007–10179	NMES + FES	C	C	C
008	N/A	X	X	X
009–10135	NMES + FES	C	Withdraw	X
010–10177	PM + FES	C	C	C
011–10064	PM + FES	C	C	C
012–10181	NMES+FES	C	C	C
013–10026	PM + FES	Withdraw	X	X
014–10149	NMES + FES	C	C	C
015–10089	NMES + FES	C	C	C
016–10140	PM + FES	C	C	C
017–10178	PM + FES	C	C	C
018–10154	PM + FES	C	C	C
019–10034	NMES + FES	C	C	C
020–10176	NMES + FES	C	C	C
021–10163	NMES + FES	Withdraw	X	X
022–10130	PM + FES	C	C	C
023–10113	PM + FES	C	C	Withdraw
024–10186	NMES + FES	C	C	C
025	N/A	x	x	X
026–10097	PM + FES	withdraw	x	X
027–10148	PM + FES	C	C	C
028–10092	NMES + FES	C	C	Withdraw
029–10180	NMES + FES	C	C	C
030–10019	PM + FES	C	C	C
031	N/A	x	x	X
032–10160	NMES + FES	C	C	X
033–10052	PM + FES	C	C	X
034	N/A	x	x	X
035	Pending randomization			
036	TBR			
037	TBR			
038	TBR			
039	TBR			
040	TBR			
041	TBR			
042	TBR			
043	TBR			
044	TBR			
045	TBR			
046	TBR			
047	TBR			
048	TBR			

*C* completed, *NA* not assigned for #008, 025, and 031, *TBR* to be recruited later

Before baseline 1, randomization was performed into NMES + FES or PM + FES groups. Post-intervention 1 (*n* = 23) was conducted after 12 weeks of intervention of either NMES or PM. Post-intervention 2 (*n* = 20) was conducted after 12 weeks of FES

**Fig. 2:**
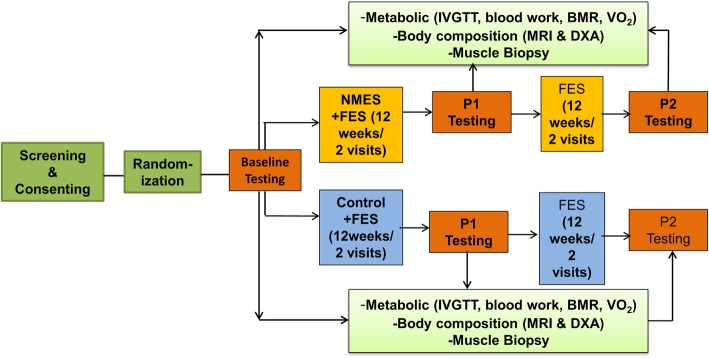
The study timeline and procedure are highlighted. After screening and consent, participants will be randomized into one of two testing groups. Each participant will undergo baseline testing (B) before beginning NMES + FES or Control + FES. Each group will then be tested for metabolic, body, and muscle composition (P1) after a 12-week period. Each group will then complete 12 weeks of FES followed by another bout of testing (P2)

Additionally, MRI scans will be obtained for trunk VAT, lower extremity skeletal muscles, and IMF CSAs [2, 3]. Participants will then be escorted to the VCU CRC and will remain in the CRC overnight for metabolic testing. This procedure will be used for the three study visits (baseline and post-interventions 1 and 2). Resting blood pressure and fasting metabolic labs will be obtained including HbA1c, as well as lipid panels, C-reactive protein (CRP), interleukin-6 (IL-6), tumor necrosis factor alpha (TNF-α), and free fatty acids (FFA). This will be followed by an intravenous glucose tolerance test (IVGTT). The vastus lateralis muscle will be biopsied to measure proteins expression and myosin heavy chain expression and to determine mitochondrial ETC and enzymatic activities. The design of the study is outlined in Fig. 2.

### Recruitment strategy

The recruitment process started in October 2015 and will end in September 2020. Forty-eight participants will be randomly assigned into either NMES + FES or Control + FES groups for 24 weeks. The participants will be matched based on level of injury (tetraplegia versus paraplegia) and time since injury (less than versus more than 10 years) using block randomization using a 2 × 2 design developed by the study statistician. Randomization was conducted using n-query computer program at the baseline before enrollment in the trial (Table 1). Current recruitment status is outlined in Table 2.

**Table 2:** The breakdown of the number of persons who have been contacted for the study, who have completed the study, or are currently actively participating in the study. All others have either failed screening or withdrawn from the study

	Study period					
	Pre-Enrolment		Enrolment	Baseline	Post-intervention	
Timepoint**	1	2			1	2
Pre-enrolment:	**536**					
Not-interested	**354**					
Expressed interest	**182**					
Phone screen Failure		**68**				
Physical screen failure		**50**				
Qualified and Enrolled		**64**				
Enrolment [1 = a +b]			**64**			
No Show ^(a)^			29			
Consented ^(b)^ [2=c +d + e + F]			**35**			
Screen Failure ^(c)^			5			
Withdrawn ^(d)^			6	3	1	2
*Completed* ^*(e)*^			21			
*Currently Active* ^*(f)*^			3			
Pending future enrollment ^(g)^			13			
Total sample size [3=b +g]			**48**			
Interventions:						
[NMES+FES]			15	14	13	12
[Control + FES]			15	13	13	12
Assessments:						
[Body Composition Variables]				X	X	X
[Metabolic variables]				X	X	X
[Muscle Biopsy variables]				X	X	X
[Torque and fatigue variables]				X	X	X

### Participant inclusion and exclusion criteria

All participants will be aged 18–65 years, men/women [24], more than one year after SCI, with body mass index (BMI) ≤ 30 kg/m^2^ [23]. Participants with traumatic motor complete or incomplete C5-L2 level of injury, the American Spinal Injury Association (ASIA) Impairment Scale (AIS) classification A, B, or C will be considered for the trial. Participants with any of the following pre-existing medical conditions will be excluded: cardiovascular disease; uncontrolled type II diabetes mellitus; uncontrolled hypertension; insulin dependence; pressures sores stage 3 or greater; hematocrit > 50%; symptomatic urinary tract infection; or participants with neck of femur or total body osteoporosis (T-score ≤ − 2.5 according to the World Health Organization guidelines.) [26].

## Interventions

### Resistance training

A recent video publication provided full details on the NMES-RT protocol [27]. Briefly, NMES will be applied to the knee extensor muscles via surface electrodes to induce concentric-eccentric actions. Two 8 × 10 cm^2^ adhesive carbon electrodes will be placed on the skin over the knee extensor muscle group. Current from the stimulator will be manually increased in 5-s intervals to evoke full knee extension against gravity followed by gradual reduction of current to induce eccentric action during relaxation. Training will be performed twice-weekly for 12 weeks. Each training session will consist of four sets of 10 repetitions of NMES with 30 Hz, 450 μs pulses, and a current sufficient to evoke full knee extension [28–30]. The first week of the RT will be conducted with no ankle weights to ensure that the knee extensor muscles can extend the weight of the lower leg against gravity [3, 23]. Once full knee extension is achieved in a sitting position, an increment of 2 lbs will be used on a weekly basis with the criteria that full knee extension should be achieved before further increases in load [21–24, 31].

### Passive range of motion for control

A member of the research team supports the leg proximal to the ankle joints and moves it from 90° knee flexion close to full knee extension. The leg will be maintained up for 5 s and returned down for 5 s. The passive movements will be repeated in the same fashion described in RT protocol: 10 reps for the right leg followed by 10 reps for the left leg for a total of four sets of 10 reps.

### Functional electrical stimulation-lower extremity cycling

A recent video publication provided full details on the FES-LEC protocol [27]. Briefly, rides will be performed with each individual using conductive adhesive gel electrodes [19, 32]. Rectangular adhesive electrodes are placed on the skin of the knee extensor, hamstrings, and gluteus maximus muscle groups. Pulse frequency will be set at 33.3 Hz, pulse duration at 350 μs, and resistance will be adjusted every 10 min to maintain a speed of 40–45 rpm (RPM). Table 3 highlights the progression of resistance in 0.5-Nm increments per 10-min stage over the course of 12 weeks. The progression in resistance was customized based on the individual’s performance riding the FES-LEC over 12 weeks. Initially, the servo-motor of the bike is permitted to assist during the warm-up phase (5 min) and during training to reduce muscle spasms. Participants are then allowed 2–3 min of rest between each 10-min stage. Time to maximum (100%) current amplitude, power, and distance covered will be recorded at the end of each stage to determine whether these parameters improved in both groups (NMES + FES versus control). The fatigue threshold will be set at 18 RPM; if RPM falls below 18 RPM, the bike will automatically shift from active to passive cycling (cool-down). During the 3-min cool-down period, participants will be passively cycled with no electrical stimulation. The cool-down period will then be followed by 5 min of recovery, during which the participant will be still connected to the bike but in a complete resting position. Heart rate will be monitored every 30 s and blood pressure every 2 min.

**Table 3:** Progression of resistance in 0.5-Nm increments per 10-min stage

	Visit 1			Visit 2			Comment
	Stage 1 (10 min)	Stage 2 (10 min)	Stage 3 (10 min)	Stage 1 (10 min)	Stage 2 (10 min)	Stage 3 (10 min)	
Week 1	1 Nm	1.5 Nm	2.0	1 Nm	1.5	2.0	If completing the 2 visits without the motor turned on, paradigm will change in week 2
Week 2	1.5 Nm	2.0	2.5	1.5 Nm	2.0	2.5	If the motor tuned on, the same paradigm will be maintained in week 3
Week 3	1.5 Nm	2.0	2.5	1.5 Nm	2.0	2.5	If completing the 2 visits without the motor turned on, paradigm will change in week 4
Week 4	2 Nm	2.5	3.0	2 Nm	2.5	3.0	And so on till week 12

The use of NMES-RT or FES-LEC may result in the following potential harms and complications:

1. Light-headedness, shortness of breath, and altered heart rate and blood pressure leading to autonomic dysreflexia. Muscle soreness of the neck, upper back, shoulders, arms, and hands;
2. Fracture of the exercising bones;
3. Autonomic dysreflexia (slow heart rate, high blood pressure, headache flushing, and sweating) which may be life-threatening;
4. Skin irritations or pressure injuries from shear stress that may lead to breaking the skin;
5. Fainting or heart attacks.

Blood pressure will be continuously monitored during exercising sessions to ensure there are no signs of orthostatic hypotension or autonomic dysreflexia; participants will be regularly checked for any changes in their physical status or health compared to their initial admission in the study and the SCI provider will be notified immediately. Before admission to the study, participants will undergo DXA scans (see below) to ensure adequate bone health—especially at the hip joints (T-scores < − 2.5 SD), distal femur, and proximal tibia (bone mineral density should be > 0.6 g/cm^2^)—to reduce the likelihood of fracture during exercising. The skin will be checked continuously on a regular basis before and after removal of the electrodes to ensure there is no redness or tendency to develop skin irritations and/or pressure injuries.

Other unanticipated adverse events will be monitored throughout the study via exams, vital signs, laboratory tests, review of medical charts, and verbal concerns voiced by the participant or an associated caregiver. Finally, data safety monitoring board of independent research investigators will annually review the study-related adverse events, adequacy of individuals’ compliance, and adherence to the protocol.

## Measurements

### Metabolic studies

#### Dietary records and basal metabolic rate

Each participant will follow a standard diet pattern during the entire period of the study (45% carbohydrate, 35% fat, and 25% protein) to balance dietary habits between both groups [23]. All participants will be asked to complete a three-day food record monitoring their energy intake each week. Previous work showed that three-day records are equivalent to five-day records in reporting caloric intake and percentage macronutrients [28]. Caloric needs will be determined using individuals measured BMR. The diaries will be evaluated weekly by the dietitian and monthly feedback will be provided via phone interview or secure email communication to ensure adherence to the recommended dietary plan throughout the study. All participants will meet with the dietitian three times: at baseline, P1, and P2.

After an overnight fast for 10–12 h, participants will be kept in a dark room for 20–30 min to attain a resting state, during which BMR will be measured by using a canopy that covers the whole head and a portable COSMED K4b2 (COSMED USA, Chicago, IL, USA). BMR and respiratory exchange ratio (RER) will be recorded by indirect calorimetry [3, 33, 34].

#### Serum total, free testosterone, IGF, FFA

Total testosterone measurements will be performed by radioimmunoassay after sample extraction and column chromatography. The interassay coefficient of variation (CV) is 12.5% or less for all quality control samples analyzed. Free testosterone concentrations will be calculated by measuring sex hormone binding globulin (SHBG) and albumin (http://www.issam.ch/freetesto.htm) [35]. Plasma IGF-I and IGFBP-3 concentrations will be measured by immunoluminometric assay (Quest Diagnostics, Madison, NJ, USA) and RIA (Diagnostics Systems Laboratories Inc., Webster, TX, USA), respectively. A total of 10 mL of blood will be collected from the indwelling venous catheter and lipid profile (HDL-C, LDL-C, total cholesterol, and TG) will be determined using standard analyses procedures.

#### Inflammatory biomarkers

Before starting the IVGTT and following a 12-h fast, blood will be collected from the indwelling venous catheter and CRP, IL-6, TNF-α, and FFA will be determined by the VCU-CRC using available enzyme-linked immunosorbent assay (ELISA) assay kits [36, 37].

#### Intravenous glucose tolerance test: primary outcome variables

An IVGTT will be used to determine insulin sensitivity and glucose effectiveness [38, 39]. Each individual will undergo an IVGTT before (baseline) and 12 weeks after interventions (Post 1 and Post 2). After a 10–12-h fast, an indwelling catheter with an intravenous saline drip (0.9% NaCl) will be placed; after 20 min of glucose injection, a bolus of insulin (0.02 U/kg) will be injected to determine insulin sensitivity. Plasma glucose will be measured by the Autoanalyzer glucose oxidase method and plasma insulin concentrations will be determined by commercial radioimmunoassay. The S_I_ (glucose disposal rate per unit of secreted insulin per unit time, i.e. insulin sensitivity) and S_G_ (glucose mediated glucose disposal rate) will be calculated from a least-squares fitting of the temporal pattern of glucose and insulin throughout the IVGTT using the MINMOD program [38, 39].

#### Oxygen uptake and energy expenditure during FES-LEC

One week before intervention (week 1), post-interventions 1 (week 14) and 2 (week 27), peak oxygen uptake (VO_2_) will be measured using a COSMED K4b2 (COSMED USA, Chicago, IL, USA) portable metabolic unit [19]. After calibration, individuals will be asked to place the mask on their face to monitor oxygen (VO_2_) and carbon dioxide (VCO_2_) production. A 3-min resting phase allows the participant to get used to breathing with the mask on while they are attached to the RT-300 bike. After the resting phase, VO_2_ will be measured during the 3-min warm-up phase, the resistance of the bike will be gradually increased by 2 Nm every 2 min until fatigue. During testing, the servo-motor will be tuned off and the cool-down phase will then be followed by the recovery phase [19].

VO_2_ and VCO_2_ will be monitored throughout exercise to determine total energy expenditure using the Weir equation. Five minutes of recovery will be recorded to determine the efficacy of each intervention on energy expenditure and substrate utilization. Heart rate (via polar HR monitor) will be recorded every 30 s and blood pressure (COSMED 740) will be recorded before, every 2 min during cycling, and for another 5 min after cycling to ensure full recovery to baseline.

### Body composition

#### BMI and anthropometrics

Each participant will be asked to empty their bladder and then will propel onto a wheelchair weighing scale to evaluate weight in kilograms. The wheelchair will be measured separately and the difference will be taken for the final weight. The height of each participant will be determined with the individual on his/her right side in the supine position. Two smooth wooden boards will be placed at the participant’s head and heels and the distance between them corresponds to the height in nearest centimeter. The BMI (kg/m^2^) will be calculated as weight (kg)/height (m^2^). Anthropometrics will be determined in duplicate by identifying the narrowest region of the trunk from sitting and lying positions. After normal expiration, a tape measure will be used around the participant’s trunk to measure waist circumference (WC) [33, 40, 41].

#### Dual energy X-ray absorptiometry (DXA)

DXA will be used to measure body composition in SCI individuals, specifically regional and total fat mass (FM) and fat-free mass (FFM). Total body and regional (lumbar spine, proximal femur, and forearm) DXA scans will be performed using a GE Lunar iDXA (Lunar Inc., Madison, WI, USA) bone densitometer at the Hunter Holmes VAMC hospital. All scans will be performed and analyzed using Lunar software version 10.5. After scanning, total and regional % FM and FFM will be determined using DXA software. The longitudinal precision of total and regional body composition using DXA was recently determined in persons with SCI [42].

#### Magnetic resonance imaging (MRI)

Skeletal muscle CSAs will be determined before (baseline) and twice after 12-week interventions (post-interventions 1 and 2) using a 1.5-Tesla GE magnet. Transaxial images, 0.8 cm thick and 1.6 cm apart, will be taken from the hip joint to the knee joint (thigh) and from knee to the ankle (leg) using the whole-body coil [19, 20, 43–46]. T1-weighted imaging will be performed using a fast spin-echo sequence to capture visceral fat images. To measure visceral adipose tissue (VAT) and subcutaneous adipose tissue (SAT), transverse slices (0.8 cm thickness) are acquired every 0.4-cm gap from the xyphoid process to the femoral heads. Images will be acquired in a series of two stacks with L4-L5 used as a separating point. Participants will be asked to take a deep breath in and hold their breath for 10–15 s to reduce the respiratory-motion artifact associated with MRI for the abdominal region [19, 29, 47].

#### Skeletal muscle torque and fatigue

Skeletal muscle torque and fatigue of the knee extensor muscle groups (left and right) will be evaluated at baseline, after 12 weeks at P1 and then 12 weeks after FES-LEC for both groups at P2 using a Biodex isokinetic dynamometer (Biodex Medical Systems, Shirley, NY, USA). Participants will be in a seated posture with the trunk-thigh angle and the knee flexed at 90° (where 0 corresponds to full knee extension). Isometric torque will be measured after adjusting the amplitude of the current to 50 and 100 mA (frequency 30 Hz and pulse duration 450 μs). Fatigue will be assessed by measuring the torque elicited at 10, 20, 30, 40, 50 60, 80, and 100 Hz before and immediately after intervention. Isokinetic torque will be measured at 60, 90, and 180 degrees/s using the same electrical stimulation protocol [19].

#### Skeletal muscle biopsy

Three biopsy samples of the vastus lateralis muscle (100 mg wet wt.) will be obtained by a 14-gauge Tru-Cut needle to compare the effects of evoked NMES + FES versus control on protein levels of IRS-1, GLUT-4, IGF-1 and PGC-1 α, AMPK, Akt and mTOR as well as the phosphorylation forms of AMPK, AKT, and mTOR. We will also test whether NMES + FES enhanced mitochondrial enzymatic activities and ETC compared to control. Skeletal muscle biopsies of the right vastus lateralis muscle will be obtained before training and after 12 and 24 weeks of interventions [48, 49].

Western blot analysis will be performed to determine the protein concentrations as previously described [50]. Briefly, proteins will be resolved by SDS-PAGE then transferred to two supported nitrocellulose membranes by wet electromembrane transfer at 110 V for 2 h and then blocked in 6% bovine serum albumin (BSA)/Tween-Tris-buffered saline (TTBS) for 1 h. Membranes will be incubated separately with the appropriate primary antibody overnight. The membranes will be washed separately (three times for 20 min) in 6% BSA/TTBS and incubated with appropriate secondary antibody in 4 mL 3% BSA/TTBS at 4 °C for 1 h. The membranes will be washed (four times for 45 min) in 6% BSA/TTBS. Proteins will be visualized using an enhanced chemiluminescence detection system (GE Lifesciences) according to the manufacturer’s instructions. Western blots will be quantified by scanning with A GS800 densitometer. Optical densities of the Western blots will be measured using image-analysis software (Molecular Analyst; Bio-Rad).

#### Mitochondrial electron transport chain activities

Samples of skeletal muscle are studied for assessment of ETC activity. Measurement of maximal enzyme activity is performed in freshly isolated detergent solubilized mitochondria using cholate as detergent. Analysis of freshly isolated samples are initially performed to assure that maximal activity of electron transport complex I is obtained. Skeletal muscle is isolated, homogenized, and a potion subjected to detergent solubilization in cholate. The enzyme activity of ETC complexes is obtained by using specific substrates and inhibitors to isolate segments of the ETC. Assays are performed in 0.1 M phosphate buffer using spectrophotometric endpoints. Complex I activity (NADH coenzyme Q reductase) is measured following the oxidation of substrate NADH to decylubiquinol, a synthetic coenzyme Q, as electron acceptor. Activity is measured in the presence and absence of the specific complex I inhibitor rotenone. In a similar manner, the activity of other complexes is measured using specific substrates, acceptors, and inhibitors. Complex II is measured using succinate as substrate and the reduction of 2,6 dichlorophenolindophenol as the electron acceptor followed as spectrophotometric indicator, with and without thenoyltrifluoroacetone as the inhibitor of complex II. Complex III activity is measured using the reduced synthetic coenzyme Q molecule decylubiquinol as substrate, oxidized cytochrome c as acceptor, and antimycin-A as the complex III specific inhibitor. The reduction of cytochrome c as acceptor is followed as the spectrophotometric endpoint. Complex IV is measured by following the first-order oxidation of added exogenous reduced cytochrome c using azide as the specific inhibitor [51]. Citrate synthase, a tricarboxylic acid cycle component enzyme, is measured as an indicator of mitochondrial mass [51–55].

### Statistical analyses

T-tests and Pearson chi-square tests will be used to compare characteristics that may act as confounding variables, including age, level of injury, time since injury, %FM, as well as the three outcome variables (Si, Sg, VO_2_) to ensure that each group is not different with respect to each variable at baseline. A repeated-measures ANOVA will be performed with glucose effectiveness as the primary study outcome and effects for treatment group (NMES + FES versus control), time (baseline and post-interventions 1 and 2), and the interaction between the two factors included in the model. Similar repeated measured ANOVA models will be fit with each of the body composition, metabolic profile, protein expression, and ETC measures (Aim 3). All statistical analysis will be performed at the 0.05 level with SAS V9.4 (Cary, NC, USA).

### Sample size

We have acquired pilot data for all outcomes of this project. Glucose effectiveness possesses the smallest effect size from baseline to follow-up with NMES-RT showing a mean baseline effectiveness of 0.01716 and post-treatment mean of 0.02258. Cohen’s *f* was found to be 0.271 using a sample variance of 0.0001 [25]. Assuming the correlation between the pre- and post-treatment measurements is 0.3735 and three total measurements are acquired, 38 individuals will need to complete the study at 90% power using a Type-1 error rate of 0.05. At least 48 participants will need to be recruited, adjusting for a 20% dropout rate. All sample sizes were calculated using the software G*Power (V3.1.9.2). A sample size of 24 was calculated using insulin sensitivity data without regard to dropout rate.

Other factors, such as insulin sensitivity and VO_2_, are considered; each representing larger effect sizes than glucose effectiveness (*f* = 0.313 and *f* = 0.432, respectively). Our study would result in a power of 97.51% and 99.82% for observing the baseline to follow-up difference between the NMES + FES and Control + FES groups, provided the correlation between baseline and follow-up measurements are 0.4 for the outcomes.

## Discussion

In the last 10 years, there have been significant advances in our understanding of the body composition changes and metabolic profile after SCI. Skeletal muscle atrophy is accompanied with infiltration of ectopic adipose tissue both in the paralyzed muscle and in trunk region that facilitates the development of central adiposity. In persons with chronic SCI, VAT and SAT are 58% and 48% greater than healthy able-bodied controls, respectively [56–58]. Ectopic adiposity is simply defined as storage of fat in non-adipose tissue sites and characterized by increasing the release of inflammatory cytokines. These changes in body fat distribution occur in conjunction with excessive production of hepatic glucose in the absence of autonomic nervous system control after SCI in those with injuries above the fourth thoracic segment [59]. The increase in VAT is negatively related to high density lipoprotein cholesterol (HDL-C) and insulin resistance, with men being 1.8–2.6 times more susceptible to accumulation of VAT than women after SCI [60]. Several anthropometric and imaging measurements have been proposed to accurately quantify changes in muscle size and visceral adiposity [61, 62]. Sumrell et al. have recommended SCI cutoffs for central adiposity that can simply be used to identify cardiometabolic risks using seated/supine circumferences in men with chronic SCI [62].

Today, exercise is still considered a feasible, accessible, and non-pharmacological approach that can overcome several of the cardio-metabolic risk factors after SCI. Upper extremity cycling exercise and/or circuit resistance training are limited to small muscle mass above the level of lesion and do not exercise large muscle groups in the lower extremities. Moreover, persons with tetraplegia (≥ C6 SCI) are less likely to benefit from upper extremity training at intensities that can improve cardio-metabolic risk parameters [63]. This renders exercise limited to use of the upper extremities as an ineffective method to attenuate the deterioration in metabolic profile and body composition after SCI. Another form of exercise is FES-LEC, which effectively restores muscle size and improve of cardio-metabolic risk factors after SCI [64, 65]. However, FES-LES is limited by low exercise intensity, oxygen uptake, and rapid muscle fatigue. To offset for these limitations, the use of hybrid exercise by combining both upper extremities and FES cycling has been shown to improve cardiovascular outcomes more than FES-LEC [66]. Arm cranking exercise (ACE) in combination with FES-LEC displayed increasing muscle oxygenation (StO_2_) at 40%, 60%, and 80% of individuals’ peak VO_2_ compared to ACE only that showed decreases in StO_2_ and FES-LEC only that had steady increases in just 60% and 80% peak VO_2_. The combination of ACE + FES-LEC demonstrates better oxygenation than either ACE or FES-LEC alone [66]. These forms of hybrid-FES training are more suited for high-intensity, high-volume exercise training than FES-cycling only and require intact upper extremity function.

There are currently no guidelines on how to maximize the benefits of FES-LEC to improve the cardio-metabolic health variables after SCI. Several strategies have been recommended to maximize the training outcomes of FES-LEC. Raising the stimulation amplitude to 300 mA has been proposed to increase the neuromuscular response and increase energy expenditure; however, this option is not FDA-approved for most of the available FES bikes [67, 68]. The maximum current on most of the commercially available FES bikes is 140 mA. Moreover, this high level of stimulation amplitude may increase the risk of developing autonomic dysreflexia. Another alternative approach is to increase the frequency of FES-LEC training to 3–7 days/week [17]. This approach is limited by poor compliance in longitudinal trials. We have studied the exercise adherence to home-based FES-LEC in 17 individuals with SCI. We have found that just maintaining a frequency of three times rather than two times/week decreased adherence from 72% in the first eight weeks to 63% in the second eight weeks [65]. A case report has shown showed that a frequency of 2–3 times per week is likely to increase adherence up to 54 months of training [69]. This is accompanied with reasonable gain in lean mass, on an average of 0.8–1.6 kg, in persons with SCI [23, 70]. Another consideration is the frequency of training and the time allowed for recovery between sessions. Our cross-sectional data showed that force remained reduced by > 20% for 48–72 h after an acute bout of FES-LEC in men with SCI [68]. This is likely caused by to exercise induced muscle damage that typically occurs as result of chronic disuse of the paralyzed muscles. It is vital to initiate appropriate mechano-stressor stimuli to optimize skeletal muscle adaptations as a large paracrine gland [71, 72]. Moreover, FES-LEC is considered an appropriate aerobic training paradigm that may stimulate an increase in mitochondrial capacity and help partitioning of substrate utilization of IMF as primary source of energy during exercise and further improved insulin sensitivity [73].

Surface NMES-RT has been shown to increase muscle hypertrophy, increase fatigue resistance, and improve oxygen reuptake in the muscles [20–25]. In a randomized clinical trial, Gorgey et al. used TRT in combination with NMES-RT to determine the impact on body composition and metabolic variables in men after SCI compared to a TRT-only control [25]. The study reports an almost 20 cm^2^ increase for whole thigh and absolute muscle CSA, in addition to an 8–12% decrease in VAT and marginal 15% increases in BMR for the TRT + RT group, with no changes in the TRT only group in thigh muscle CSA or BMR. The increases in lean mass and BMR combined with the decreases in VAT content after TRT + RT may improve cardiometabolic health, insulin sensitivity, and prevent obesity [25]. Moreover, we and others have shown that NMES-RT is accompanied with decrease infiltration of IMF, reduce peripheral muscle fatigue by 30%, increase muscle quality and slowness of rise time, and increase mitochondrial activity after SCI [74, 75].

### Limitations

Dietary intake may confound effects of the training and increased muscle mass. However, a recent trial showed that protein intake over an eight-week period would not trigger increase in muscle size in persons with SCI [76]. The dietary records are evaluated on a weekly basis to monitor caloric intake. Any intake that exceeds 300–500 kcal/week above their baseline BMR will be flagged. Another limitation is that close to 20% of the population may not benefit from NMES/FES applications, particularly those with lower motor neuron injury and denervation of skeletal muscles, thus limiting eligibility for enrollment and reducing the pool of patients who may benefit from NMES/FES as a therapy. Another limitation is those who do not respond to NMES due to lower motor neuron denervation (LMN) or intolerance to electrical stimulation [77]. Currently, a clinical trial is underway to test the efficacy of using long pulse width stimulation to restore muscle size in those with LMN after SCI [77].

We proposed a two-center study to facilitate recruitment of 48 participants. We have included an age range of 18–65 years, women, and those with AIS C, provided a wheelchair is their primary method of mobility, who possess no functional movement sublesionally and use non-weight bearing activities only. The feasibility of this approach may be called into question because any benefits may be lost with the cessation of training. Our protocol emphasizes long-term compliance and adherence to maximize benefits. We have tested the feasibility of home-based NMES-RT over an eight-week period utilizing video-telecommunication service. The result showed that the trained knee extensor muscle CSA increased by 18% compared to the controlled limb [78]. This may serve as a continuation of the current trial and bases for establishing future home-based clinical trials to maximize recruitment and reduce the cost associated with travel time.

Previously, the barriers of commitment to long-term intervention have been highlighted [79]. One of these barriers is transportation, which remains of a great concern regarding retention in the study. To further alleviate transportation burdens, a research technician has been budgeted to escort participants in a VA van to certain locations for study measurements.

In conclusion, the current clinical trial endeavors to increase the cardio-metabolic benefits of FES-LEC through the addition of NMES-RT. This may result in improvement of aerobic fitness and metabolic health and reduce the severity of chronic secondary health-related consequences. The study will provide mechanistic insights regarding molecular underpinnings of the benefits of the two different paradigms of electrical stimulation to chronically paralyzed muscles. One paradigm is mimicking RT using a weight-lifting approach and the other generates low tension stimulation mimicking aerobic-type training. It will be of interest to our population to understand the additive effects of both approaches on the cardio-metabolic outcome variables in persons with SCI. This could be simply integrated as an effective rehabilitation intervention in the clinical setting shortly after completion of the study.

## Trial status

The trial has been active and open for enrollment since August 2015 with anticipated completion date of September 2020. The study was approved by the local ethics committee at McGuire VA Medical Center and Virginia Commonwealth University. The trial is registered in clinicaltrials.gov, no. NCT02660073. Protocol version-3 dated 25 November 2015. Recruitment started August 2015 and will end March 2020.

## Data Availability

The data will not be publicly available and only data will be available upon written request to the PI. **Confidentiality, Storage, and Sharing of Data** Participants will be assigned a unique study number upon entrance into the study. All forms will be coded with this number rather than the patient’s name, social security number, or other identifying information. Participant identifiers will be available to the PI and authorized research coordinator via a password protected database and/or coding sheets kept in locked areas of the research personnel offices. The principal investigator and study personnel will keep a master. The PI, or designee, may share data collected through study screening and/or participation with the participant or their primary care provider if there is a likelihood that the participant could benefit medically or otherwise from having the information and subjects will be notified of this through the informed consent process and in the written consent document. **C1. Data Manager** The data manager’s duties will include continuous data coordination, management, and monitoring for the project. The data manager is responsible for the development and maintenance of a comprehensive database and in the preparation of data for statistical analysis for the study. Also, the data manager will be responsible for supervising and facilitating electronic (and possibly manual) data entry and data collection tools, including the physical handling of paper data forms and any other collection devices. Also, the data manager will assist the biostatistician in testing data forms and transferring data out of the database for preparation and analysis. **C2. Database Management, Data Monitoring, and Quality Control** Subject list that links the subject ID number with personal identifiers. This master list and the study documents and research records will be maintained in separate locked cabinets in a locked research office. All study records will be maintained indefinitely. Individuals from the local institutional review board (IRB) may look at these records as part of their duties. Confidentiality of these records will be protected to the extent possible under existing regulations and laws. Participant’s name and other protected health information will not appear in any published paper or presentation related to this study. It should be noted that representatives of the McGuire Research Institute (MRI) local IRB are eligible to review research records as part of their responsibility to protect human subjects in research. Additionally, research records may be reviewed by appropriate federal agencies, such as the Food and Drug Administration (FDA), as required by law. Data entry will be facilitated by the development of an MS Excel data entry sheets. The MS Excel front end has built-in first-level automatic checks to ensure data integrity and validity regarding data type and range, as well as data completeness. Summary and raw data reports will be integral parts of the database design to help the investigators routinely inspect entered data. Each participant will have a summary excel sheet coded by his/her assigned study ID code. The excel sheet will be composed of three tabs (baseline, post-intervention 1 and post-intervention 1). After subject’s data has been entered into the database, a 100% data audit is performed to assure accuracy of the entered and constructed data. Any systematic problems should be detected than 1% error rate. If, however, the error rate is larger than 1%, the effected subset will be audited on a more frequent basis until the problem is resolved. Finally, the original databases will be housed on the VA password-secured drive that only the PI and his research coordinator will have access to it. The data manager is tasked to export data from the back-end and store data in an “analysis ready” format for subsequent data analysis using SPSS 24 statistical analysis packages. Technical support of the database applications will be provided on an ongoing basis throughout the study through the manuscript preparation phase. **C4. Data Flow** Data will be obtained using traditional data collection forms and exported electronic Excel sheet files format that automatically generated during for energy expenditure, body composition assessment using DXA and MRI as well as secured templates for IVGTT. Duplicates of the traditional data collection forms will be securely sent to the Data Management Center and the originals will be securely stored at McGuire VA Hospital. Data collected through traditional data collection forms will be entered by hand into MS Excel sheet by the research study coordinator. Finally, data will be copied onto a CD with no personal identifiers or a secure USB drive and securely sent to the Data Management center where they will be uploaded and imported into Microsoft Excel (original files will remain at McGuire). The data will be stored on a secure VA flash drive with incremental backups. **C5. Data Center Confidentiality and Security** All database applications will be password-protected to ensure security of entered data and housed in the McGuire VA Medical Center servers located behind the firewall. Only the PI and other approved personnel will have access to the data. There will be several levels of security and access will depend upon the personnel’s need. Exported data will not contain any information regarding patient identity (i.e. name, social security number, address, birthday, medical record number, and any other identifying information). Subject ID or similar naming convention will identify each participant, thus ensuring strict confidentiality of all study data. The server that the database is maintained on will also have its own level of security with access to the data only by password, thus ensuring two levels of security. The data manager will be responsible for keeping a (local) redundant back-up of all active databases. All study data will be backed-up on a weekly basis onto CD-ROM or external hard drive. The copies will be kept in a fireproof safe in the data manager’s office.
